# Validity of summing painful joint sites to assess joint-pain comorbidity in hip or knee osteoarthritis

**DOI:** 10.1186/1471-2474-14-234

**Published:** 2013-08-09

**Authors:** Liseth Siemons, Peter M ten Klooster, Mart A F J van de Laar, Cornelia H M van den Ende, Thomas J Hoogeboom

**Affiliations:** 1 Department of Psychology, Health & Technology, Arthritis Center Twente, University of Twente, Enschede, The Netherlands; 2 Department of Rheumatology, Arthritis Center Twente, Medisch Spectrum Twente, Enschede, The Netherlands; 3 Department of Rheumatology, Sint Maartenskliniek, Nijmegen, The Netherlands; 4 Department of Epidemiology, CAPHRI, CCTR; Maastricht University Medical Centre +, Maastricht, The Netherlands; 5 Department of Psychology, Health & Technology, Faculty of Behavioural Sciences, University of Twente, PO Box 217, 7500, AE, Enschede, the Netherlands

**Keywords:** Hip or knee osteoarthritis, Joint-pain comorbidities, Rasch analysis, Sum score, Validity

## Abstract

**Background:**

Previous studies in patients with hip and knee osteoarthritis (OA) have advocated the relevance of assessing the number of painful joint sites, other than the primary affected joint, in both research and clinical practice. However, it is unclear whether joint-pain comorbidities can simply be summed up.

**Methods:**

A total of 401 patients with hip or knee OA completed questionnaires on demographic variables and joint-pain comorbidities. Rasch analysis was performed to evaluate whether a sum score of joint-pain comorbidities can be calculated.

**Results:**

Self-reported joint-pain comorbidities showed a good fit to the Rasch model and were not biased by gender, age, disease duration, BMI, or patient group. As a group, joint-pain comorbidities covered a reasonable range of severity levels, although the sum score had rather low reliability levels suggesting it cannot discriminate well among patients.

**Conclusions:**

Joint-pain comorbidities, in other than the primary affected joints, can be summed into a joint pain comorbidity score. Nevertheless, its use is discouraged for individual decision making purposes since its lacks discriminative power in patients with minimal or extreme joint pain.

## Background

Previous studies in patients with hip and knee osteoarthritis (OA) advocated the relevance of assessing the number of painful joints, other than the primary hip or knee joint, in both research and clinical practice [1-3]. Joint-pain comorbidities are related - both cross-sectionally and longitudinally - to unfavourable health outcomes, including a higher physical and psychological burden, more severe fatigue, and worse health-related quality of life [2,3].

To date, the use of joint counts to indicate the level of joint-pain comorbidity in people with knee or hip OA is common [2-5]. By simply summing up the number of joints that are indicated as painful for more than half of the time during the last month, a fast and easy impression of a patient’s overall degree of joint-pain comorbidities is obtained. To determine painful joints, researchers often use written questions or a manikin. Both methods are deemed valid, although the use of a manikin is known to result in a higher prevalence of musculoskeletal pain [6]. Remarkably, however, it remains unclear whether this simple summation of painful joints is actually justified. One could make the argument that this is likely not the case, as summing joints would mean that each of the joint scores assess the same underlying construct and that all joints are of equal importance (i.e. have equal weights) [7].

Therefore, the aim of this study was to examine the validity of summing painful joint sites in people with hip or knee OA.

## Methods

### Patients and measures

Data were used from a previous study, which examined the prevalence of joint-pain comorbidities in patients with a physician-based diagnosis of hip or knee OA [2]. The patients were included if they were 18 years or older and visited an orthopaedic surgeon at the Sint Maartenskliniek (Netherlands) for a new episode of complaints due to hip or knee OA (somewhere between June and October 2009). Patients concurrently suffering from an underlying rheumatic disease were excluded.

At baseline, information was gathered on gender, age, body-mass index (BMI), and disease duration. In addition, the patients received a questionnaire within 14 days after their visit, assessing their joint pain at 19 joint sites, including the hands, wrists, elbows, shoulders, cervical spine, thoracic spine, lumbar spine, hips, knees, ankles, and feet. Patients had to indicate whether they experienced symptoms from particular joints and whether these symptoms were present for more than half of the time during the last month. If both questions were positive, the joint was counted as being painful.

The study was approved by the Institutional Review Board of the University Medical Centre, Nijmegen and all patients enrolled in the study gave informed consent.

### Statistics

#### Rasch analysis

Whether the joint-pain comorbidities can simply be summed up was evaluated using Rasch analysis. A feature of the Rasch model is that it assumes all items to be equally discriminating [8]. As a result, a good fit between the Rasch model and the data indicates that individual joint scores can be summed up to obtain a total score of joint-pain comorbidities during the past month [7].

In order to perform Rasch analysis, the data must conform to a number of underlying assumptions of the Rasch model [8-10] including unidimensionality, model fit, and local independence.

The first assumption, unidimensionality, was tested with a principal component analysis of the tetrachoric correlation matrix in SPSS Statistics 18.0, using oblimin rotation. The scale was assumed to be sufficiently unidimensional (i.e. there is one dominant underlying factor) if the ratio of the first and second eigenvalue was >3 : 1 [11].

The second assumption concerns the model’s ability to reflect the true relationship among the underlying construct and the item responses [8-10]. This was tested by evaluation of the mean square Infit (Infit MNSQ) and mean square Outfit (Outfit MNSQ) fit statistics. Mean square values show the ratio between the observed and predicted variance, with an expected value of 1.0 [12]. Corrected for the sample size of 401 patients, the Infit and Outfit ranges required for a good fit are 0.90-1.10 and 0.70-1.30 respectively [13]. Higher values show unexpected responses (noise) or might point to multidimensionality. Lower values point to item redundancy, meaning that the information provided by the item overlaps with the information provided by other items [12,14,15].

The final assumption assumes that the items are not further associated with each other once the Rasch factor is taken into account. Violation of this assumption might point to response dependency (e.g. overlapping items in the scale) or to multidimensionality of the scale [9,12]. Items were assumed to be locally dependent if the residual correlation between two items was >0.5 [12].

In case the Rasch assumptions were satisfied, joints were checked for differential item functioning (DIF). DIF is present when subgroups of patients with similar levels of the measured underlying construct (i.e. the degree of joint-pain comorbidity as measured by summing the number of painful joints) give different responses (i.e. painful or not painful) to a specific joint [9,10]. DIF was tested for age, gender, BMI, disease duration, and patient group. Subgroups of age (≤58 and >58) and disease duration (≤10 years and >10 years) were created by splitting the group at the median. BMI subgroups were created by splitting the group at the BMI cut-off point for overweight (BMI≥25). Patient groups were formed by separating the knee OA patients from the hip OA patients. In case a patient experienced both knee as well as hip pain, the patient was classified based on their primary complaint.

Finally, the performance of the sum score was examined by evaluating its test information function with associated reliability levels; showing whether precise and reliable joint-pain comorbidity scores can be obtained across the range of joint pain comorbidity severity. Reliabilities >0.7 were deemed acceptable for group use [9]. In addition, the higher the test information, the better the test will be able to discriminate among individuals [10].

Rasch analyses were performed using Winsteps, version 3.65 (Winsteps, Chicago, IL, USA).

## Results

### Patients

Data were collected from 401 patients (58% [n=231] women and 42% [n=170] men), of which 71% were referred for knee OA and 29% for hip OA. The average (SD) age of the study sample was 58 (13) years, with an average (SD) body mass index of 27 (5) kg/m^2^. The majority of the participants had a K/L grade of ≥ 2 and a disease duration of >5 years, respectively 76% and 61%. Mean (SD) pain levels were 45 (21) points for the hip group and 50 (20) points for the knee group, as measured with the Hip disability and Osteoarthritis Outcomes Score and Knee Injury and Osteoarthritis Outcome Score respectively [16,17]. More detailed sample characteristics have been described elsewhere [2,18].

### Rasch analysis

The test of unidimensionality showed that the ratio between the first and second eigenvalue was 9.701 : 2.117, which is >3 : 1. Thus, the 19 items formed a sufficiently unidimensional scale for Rasch analysis.

The results of the Rasch analysis showed an acceptable fit of the comorbid joints (i.e. other than the primary affected joints) to the Rasch model (Table 1). Infit MNSQ was <0.90 or >1.10 for 7 out of the 19 joints and Outfit MNSQ was <0.70 or >1.30 for only 5 joints. Additionally, none of the items showed DIF and local independence of the items was supported by low inter-item residual correlations (<0.49).

**Table 1:** Rasch fit statistics of all 19 items (N=401), with hip scores removed for hip patients and knee scores for knee patients

**Joint**	**Measure**	**Fit**				**DIF**				
	**Logits**	**Infit MNSQ**	**Infit ZSTD**	**Outfit MNSQ**	**Outfit ZSTD**	**Age**	**Gender**	**BMI**	**Disease duration**	**Patient group**
Lumbar spine	−2.02	**1.23**	3.5	1.28	2.3	−0.73	0.26	0.20	0.14	1.14
Left knee	−1.45	**1.24**	1.8	1.21	1.0	1.03	1.12	0.45	−0.70	N/A
Right knee	−1.11	1.02	0.2	1.06	0.3	−1.11	−0.31	0.30	−0.10	N/A
Neck	−0.90	1.00	0.1	1.04	0.4	−0.17	0.02	−0.84	0.07	−0.11
Right shoulder	−0.46	1.04	0.4	1.01	0.1	−0.17	−0.18	−0.16	0.11	0.04
Left shoulder	−0.21	**0.87**	−1.2	0.72	−1.6	0.21	−0.32	−0.05	−0.06	−0.15
Right hand	−0.10	0.95	−0.4	0.99	0.0	0.99	−0.53	−0.05	−0.23	−0.65
Left foot	0.06	0.94	−0.5	0.89	−0.4	0.19	−0.10	0.37	0.11	−0.45
Right hip	0.07	1.02	0.2	1.04	0.2	−0.16	0.03	0.17	0.59	N/A
Left hip	0.07	**1.16**	1.2	**1.38**	1.5	−0.84	−0.49	−0.30	0.45	N/A
Right foot	0.10	0.94	−0.5	0.77	−1.1	0.11	−0.03	−0.12	−0.46	0.08
Left hand	0.24	1.05	0.4	1.09	0.4	1.04	−0.26	−0.06	−0.28	−0.78
Thoracic spine	0.48	1.09	0.7	0.85	−0.5	−0.22	0.11	0.19	0.45	0.85
Right ankle	0.54	0.99	0.0	0.98	0.0	−0.53	0.19	−0.25	−0.60	0.69
Left ankle	0.59	**0.89**	−0.7	**0.67**	−1.3	−0.21	−0.77	0.10	−0.21	−0.69
Right wrist	0.65	**0.79**	−1.3	**0.52**	−1.9	0.38	−0.14	0.28	0.02	−0.62
Left wrist	0.71	**0.87**	−0.7	**0.63**	−1.3	1.08	−0.03	0.00	0.04	−0.19
Right elbow	1.11	1.09	0.5	**1.33**	0.9	−0.46	1.57	0.66	0.38	−0.99
Left elbow	1.65	0.95	−0.1	0.88	−0.1	−0.05	0.92	0.32	0.20	−1.02

Infit MNSQ: mean square Infit, Outfit MNSQ: mean square Outfit, ZSTD: standardized as a z-score, DIF: differential item functioning, BMI: body-mass index, N/A: not available. Bold values show either Infit MNSQs or Outfit MNSQs outside their specified range or show the presence of differential item functioning (DIF). DIF would have been significant when: *p*< (0.05/(5*19))= 0.000526 (corrected for sample size) AND when the absolute difference between the two difficulty parameters was ≥0.5 logits. However, no significant DIF results were found in this study.

Although some Infit and Outfit values fell outside the specified range for a number of joints, these analyses were based on very strict sample size corrected Infit and Outfit statistics. A more commonly applied, less strict, rule of thumb is that both the Infit and the Outfit MNSQ are allowed to be somewhere between the 0.7 and 1.3. When these values were held against the data, fit was even better with none of the joints showing Infit MNSQ values <0.70 or >1.30 and only 5 joints showing Outfit MNSQ values <0.70 or >1.30.

**Figure 1:**
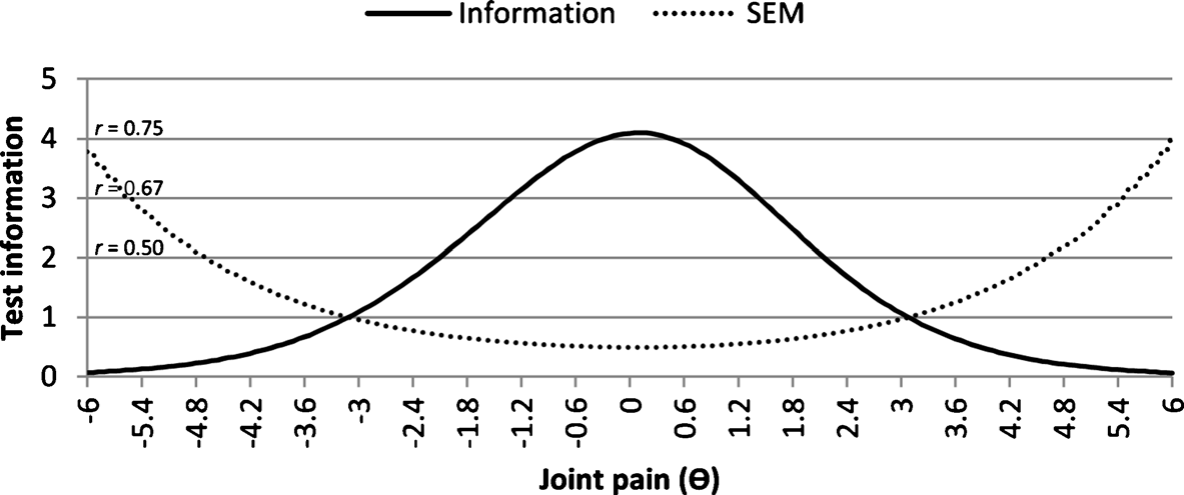
**Test information function of the 19 items, with hip scores removed for hip patients and knee scores for knee patients.** Its associated reliability level (*r*) and standard error of measurement (SEM) are included and ϴ indicates the latent variable (standardized to a logit score of 0). Higher values of ϴ indicate a higher number of painful joints. Logit values of -3, 0, and 3 correspond approximately to total comorbidity scores of 1, 9-10, and 18, respectively.

The test information function (Figure 1) shows that joint-pain comorbidities covered a reasonable range of joint-pain comorbidity levels. However, its measurement reliability was relatively low with a maximum value of 0.76. Reliability was only above the minimally required value for group use, *r* > 0.70, within a range of -1.04 to +1.18, corresponding to the presence of 5 to 14 painful joints. Although this indicates that the instrument can discriminate well between patients within these wide range of joint scores, it only covered 11.2% of the patient sample since most patients (86.8%) had less than 5 painful joints. The instrument’s discriminative power for this large majority of patients with few complaints is much lower, diminishing the instrument’s usefulness.

## Discussion

The past decades show a marked increase in item response theory applications within the rheumatic field [19]. Item response theory offers a powerful framework for developing and evaluating patient-reported and clinical measures, item banks, or computerized adaptive tests. In addition, Rasch analysis can be used for examining the validity of a scoring algorithm like the summation of pain scores of individual joints. This study confirms that joint-pain comorbidities, in other than the primary affected joints, can be summed into a joint pain comorbidity score, as frequently used in previous studies in order to determine its association to patients’ health states [2-4,20]. The absence of DIF across age, gender, disease duration, BMI, and patient group indicates that this measure can validly be used in research. However, even though the analyses confirmed that the instrument can measure a reasonable range of pain comorbidities, the instrument’s reliability was rather low and it lacks discriminative power in patients with minimal or extreme joint pain. Therefore, its use is discouraged for individual decision making purposes in clinical practice.

Comorbidity is very common in people with OA [21,22] and is linked to the burden of illness [23]. A better understanding of comorbidity in OA patients might enable us to define new strategies to manage the OA symptoms. Joint pain comorbidity can play an important role in OA since previous studies already demonstrated that higher joint counts in people with OA are associated with less favorable health states [2,3] and worse outcomes after total knee replacement surgery [4]. Now that we demonstrated that joint counts can be validly used in medical research concerning patients with hip and/or knee OA, future studies should further explore joint pain impact and perhaps its manageability [24].

## Conclusions

The results of this study showed that it is valid to sum the number of painful joint sites to assess joint-pain comorbidity in hip or knee osteoarthritis. However, prudence is in order when using this instrument for treatment decisions on an individual level.

## Abbreviations

OA: Osteoarthritis; BMI: Body mass index; MNSQ: Mean square; DIF: Differential item functioning.

## Competing interests

None of the authors have financial, commercial, or other associations that might pose a conflict of interest in connection with the work.

## Authors’ contributions

LS was responsible for the conceptualization of the manuscript. EVDE and TH were responsible for data collection. PTK, TH, EVDE, and MVDL supervised the whole study and the interpretation of the results. All authors critically evaluated the manuscript, contributed to its content, and approved the final version. LS and TH take responsibility for the integrity of the work as a whole, from inception to finished article (L.siemons@utwente.nl, thomashoogeboom@gmail.com).

## Pre-publication history

The pre-publication history for this paper can be accessed here:

http://www.biomedcentral.com/1471-2474/14/234/prepub
